# Dr. Hilles Wynkoop

**Published:** 1909-05-22

**Authors:** 


### Obituary.

Dr. Hilles Wynkoop, the well-known New York surgeon, died last week in that city. The cause of his death is stated to have been appendicitis. He was 66 years of age.

